# Sleep Duration and Waking Activities in Relation to the National Sleep Foundation’s Recommendations: An Analysis of US Population Sleep Patterns from 2015 to 2017

**DOI:** 10.3390/ijerph18116154

**Published:** 2021-06-07

**Authors:** Michael Osei Mireku, Alina Rodriguez

**Affiliations:** 1 School of Psychology, University of Lincoln, Lincoln LN6 7TS, UK; 2 Lincoln Sleep Research (LiSReC), University of Lincoln, Lincoln LN6 7TS, UK; 3 Department of Epidemiology and Biostatistics, School of Public Health, Imperial College London, London W2 1PG, UK; a.rodriguez@imperial.ac.uk; 4 Centre for Psychiatry, Wolfson Institute of Preventive Medicine, Queen Mary University of London, London EC1M 6BQ, UK

**Keywords:** sleep duration, waking activities, time use, suboptimal sleep, excessive sleep, recommendations

## Abstract

The objective was to investigate the association between time spent on waking activities and nonaligned sleep duration in a representative sample of the US population. We analysed time use data from the American Time Use Survey (ATUS), 2015–2017 (*N* = 31,621). National Sleep Foundation (NSF) age-specific sleep recommendations were used to define recommended (aligned) sleep duration. The balanced, repeated, replicate variance estimation method was applied to the ATUS data to calculate weighted estimates. Less than half of the US population had a sleep duration that mapped onto the NSF recommendations, and alignment was higher on weekdays (45%) than at weekends (33%). The proportion sleeping longer than the recommended duration was higher than those sleeping shorter on both weekdays and weekends (*p* < 0.001). Time spent on work, personal care, socialising, travel, TV watching, education, and total screen time was associated with nonalignment to the sleep recommendations. In comparison to the appropriate recommended sleep group, those with a too-short sleep duration spent more time on work, travel, socialising, relaxing, and leisure. By contrast, those who slept too long spent relatively less time on each of these activities. The findings indicate that sleep duration among the US population does not map onto the NSF sleep recommendations, mostly because of a higher proportion of long sleepers compared to short sleepers. More time spent on work, travel, and socialising and relaxing activities is strongly associated with an increased risk of nonalignment to NSF sleep duration recommendations.

## 1. Introduction

Optimal sleep duration is crucial as both short and long sleep duration are associated with physical and mental health problems [1,2,3]. For example, sleep duration that is considered to be too short has been linked with an increased risk of obesity [4,5], hypertension [6], cardiovascular disease [7], and type II diabetes [8], whereas sleep duration that is too long has been associated with an increased risk of poor health-related quality of life [9], multiple sclerosis [10], cardiovascular disease, and stroke [11]. However, the thresholds for understanding optimal sleep durations are fuzzy and vary across studies [8,12,13], impeding comparisons, especially considering that the physiological demand for longer sleep declines with age [14]. In response to this concern, the United States National Sleep Foundation (NSF) defined age-specific recommendations for sleep duration in 2015 based on population-based data and index literature published between 2004 and 2014 [15]. These sleep durations guidelines provide detailed recommendations for nine age groups (Table 1).

Here, we use these recommended guidelines to study sleep patterns in a population-based sample of over 31,000 US residents from the American Time Use Survey (ATUS) from 2015 to 2017 [16]. As previously indicated [17], habitual sleep duration represents a trade-off with waking activities. While the nature of waking-related activities, such as the stressfulness of work, type of diet, and the intensity of physical activity, can influence sleep [18,19,20], the time spent on these waking activities also competes with sleep duration in a 24 h world [17,21], warranting the need to maintain sleep and wakefulness [22].

Previous work using the ATUS examined sleep–wake time; however, the authors could not compare the prevalence of sleep duration to the NSF guidelines as these were published eight years later. Because waking activities are likely to have changed since the original report was published in 2007 [17], we provide an update and extend the analysis by comparing reported sleep duration with NSF age-specific guidelines, while examining sociodemographic factors. We also examine how time spent on waking activities relates to the aligned sleep duration with NSF guidelines.

## 2. Materials and Methods

### 2.1. American Time Use Survey (ATUS) Participants

The ATUS is a federally administered annual survey sponsored by the Bureau of Labor Statistics and conducted by the US Census Bureau. The survey includes a nationally representative sample of US residents, aged 15 years and over, who are neither residents of nursing homes or prisons nor in active military duty. The sample frame for the ATUS is provided by the Current Population Survey (CPS), a continuous survey of representative sample households. All members of an eligible household have equal probability of being selected into an ATUS household if they meet the eligibility criteria. To ensure adequate representation, the ATUS oversamples eligible households with Hispanics and non-Hispanic Black people, households with children, and weekend data collection. The primary objective of the ATUS is to measure how US residents spend their time on a typical day. The ATUS therefore captures a 24 h time activity diary recall, to 1 min resolution, of eligible respondents starting at 4 a.m. the previous day and ending at 4 a.m. on the interview day via a computer-assisted telephone interview lasting between 15 and 20 min per respondent [16]. The designated person who consents to participate is given USD 40 incentive for their time.

### 2.2. ATUS Variables and Coding

Detailed activities are coded and classified into 17 major categories labelled from 01 to 17. Each category is further classified into two-tiered subcategories, each labelled with two digits. For example, time spent sleeping (t010101) is a subcategory of sleeping-related activities (0101), which is also a subcategory of personal care activities, a major category (01). Thus, each pair of digits indicates the first, second, and third tiers in order. Due to the complexity of coding daily activities, coders follow a strict flowchart to help to categorise an activity into this three-tier coding system. Detailed information about coding, categorisation, verification, and adjudication of activities is available in the ATUS User’s Guide [16].

### 2.3. Data Processing

The ATUS has been continuously run since 2003. For this article, only pooled data from 2015 to 2017 comprising 31,621 respondents from a designated sample of 69,838 were used. The configuration of the ATUS database and corresponding survey response rates from 2015 to 2017 is presented in Table 2.

### 2.4. Data Analysis

First, we focused on sleep duration and waking activities and mapped these to the NSF sleep guidelines in the population. An adjusted Wald test was used to compare average sleep duration between groups of categorical variables. For this part of the analysis, we generated 4 age categories to reflect age groups with different NSF sleep recommendations, i.e., <18 years, 18 to 25 years, 26 to 64 years, and ≥65 years (see Table 1). We then calculated the prevalence of sleep duration for the weighted proportion of the US population to the age-specific NSF sleep recommendations. In this way, we mapped sleep duration according to its alignment with the NSF recommendations as appropriate recommended duration (AR), appropriate but short (AS), appropriate but long (AL), not recommended: too short (NRS), and not recommended: too long (NRL). We then calculated the weighted average sleep duration per year for weekdays and weekends for each age category.

We used the chi-square test of independence to compare the weighted proportion of the five alignment categories by sociodemographic variables. To account for the survey nature of the data, a second-order Rao and Scott correction was used to generate non-integer degrees of freedom, which were then used to compute the corrected F-statistic and corresponding *p*-values [18]. The sociodemographic variables considered in the inferential analysis include age, race, highest level of education, employment status, family income, marital status, and number of household children under 18 years. Next, we investigated the difference in the time spent on waking activities to the NSF recommendations by running weighted simple linear regression models. The AR category was used as the reference of the NSF sleep adherence variable in the regression models. Further, models were adjusted for all the sociodemographic variables in weighted multiple linear regressions. The regression coefficients compared the adjusted time spent on waking activities by more than 15 min between individuals meeting and those not meeting the recommended guidelines for sleep duration.

We used the chi-square test of independence to compare the weighted proportion of the five alignment categories by sociodemographic variables. To account for the survey nature of the data, a second-order Rao and Scott correction was used to generate non-integer degrees of freedom, which were then used to compute the corrected F-statistic and corresponding *p*-values [18]. The sociodemographic variables considered in the inferential analysis include age, race, highest level of education, employment status, family income, marital status, and number of household children under 18 years. Next, we investigated the difference in the time spent on waking activities between each of the sleep duration categories and the recommended category by running weighted simple linear regression models. The AR category was used as the reference of the indicator NSF sleep adherence variable in the regression models. Further, models were adjusted for all the sociodemographic variables in weighted multiple linear regressions. The regression coefficients compared the adjusted time spent on waking activities by more than 15 min between individuals meeting and those not meeting the recommended guidelines for sleep duration.

All analyses were performed using Stata/SE 15.1 for Windows (StataCorp LP, TX, USA). Statistical significance was defined as *p* < 0.05. To correct oversampling in the ATUS dataset, representative estimates of time spent on different activities were calculated using sampling and replicate weights provided by the ATUS. Representative average estimates were calculated by making the survey design variables identifiable (using *svyset*, StataCorp LP, TX, USA) and using the balanced repeated replicate (BRR) weights and a Fay’s adjustment of 0.5 [17]. For this article, ATUS respondents who were recruited on holidays were excluded from analyses (*N* = 456).

## 3. Results

The sociodemographic characteristics of the ATUS respondents are presented in Table 3. Most of the population were women (51.7%), white (65.7%), and had no child in the household (61.9%).

Weighted average sleep duration on weekdays and at weekends by sex from 2015 to 2017 is presented in Table 4. In general, average sleep duration was longer at weekends (*M* = 9.3 h, *SE* = 0.02 h) than on weekdays (*M* = 8.5 h, *SE* = 0.02 h). The adjusted Wald test showed that the difference between average weekend and weekday sleep duration was significant, *F*(1, 159) = 875.4, *p* < 0.001). However, there was no difference in average sleep duration, either on weekdays, at weekends, or in total, across the ATUS recruitment years 2015–2017. When comparing average sleep duration by sex, we observed that, in general, women had 9 min longer average sleep duration than men, *F*(1, 159) = 24.5, *p*< 0.001). After stratifying the analysis by weekday/weekend, this difference only remained for weekday sleep (mean difference, *MD* = 12 min, *F*(1, 159) = 20.7, *p* < 0.001)), but not weekend sleep.

**Figure 1:**
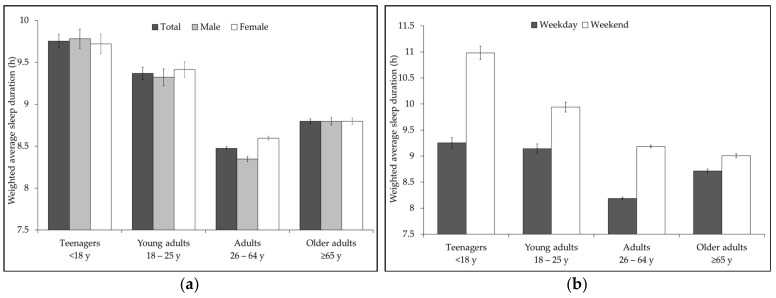

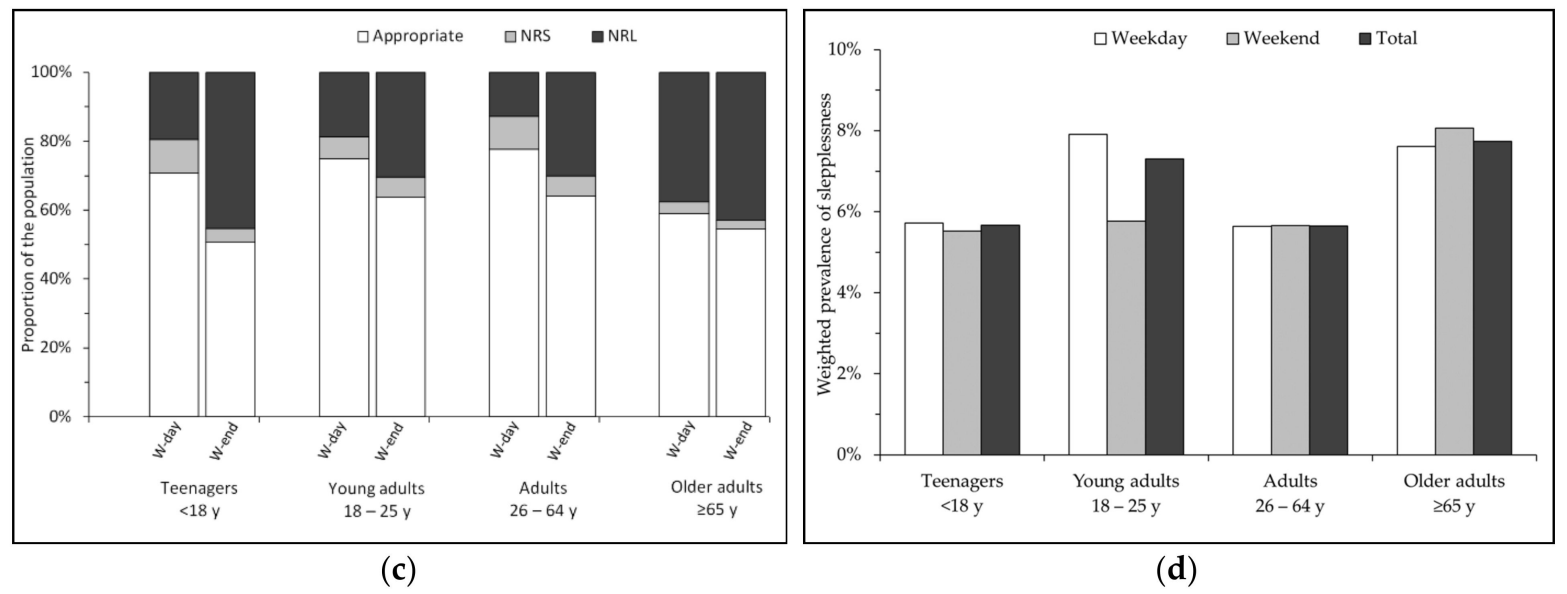
Sleep characteristics of ATUS respondents: (**a**) Average sleep duration by sex and age categories. Teenagers’ age category includes 15- to 17-year-olds; (**b**) average weekday and weekend sleep duration by age categories; (**c**) distribution of the population by three sleep duration alignment categories (appropriate, ARNRS—not recommended: too short; NRL—not recommended: too long) on weekdays (w-day) and at weekends (w-ends); (**d**) proportion of sleeplessness on weekdays and at weekends by age categories.

Figure 1a displays the average sleep duration in hours by age and sex. Sleep duration was highest among teenagers (*M* = 9.8 h, *SE* = 0.08 h) and lowest among the adult group (*M* = 8.5 h, *SE* = 0.02 h). The difference in sleep duration between age groups after pairwise comparison was consistently statistically significant among men (all Šidák-adjusted *p*s < 0.001) but not among women. For women, average sleep duration was significantly different between all the age groups pairs (all Šidák-adjusted *p*s < 0.001) except between teenagers and young adults. Likewise, weekday average sleep duration was significantly different between all the age groups pairs (all Šidák-adjusted *p*s < 0.001) except between teenagers and young adults (Figure 1b). In total, the proportion of population who slept the NSF recommended duration was 45.3% on weekdays and 32.8% at weekends (Table 5). The proportion of the population in the five sleep recommendation categories was significantly different on weekdays and at weekends, *F*(3.8, 608.1) = 248.4, *p* < 0.001. Results for the weighted proportion of sleep duration on weekdays to weekends showed that sleeping duration was outside the recommended range and was too long (NRL) at weekends than on weekdays irrespective of the age category (all *p*s < 0.001) (see Figure 1c). The weighted proportion of ATUS respondents reporting less than the recommended amount (NRS) was 6.3%. Less sleep duration was more prevalent in the older adult population (7.7%) and lowest among the adult population (5.6%). Across the different age categories, prevalence of sleeplessness on weekdays was not different from weekends (Figure 1d).

**Figure 2:**
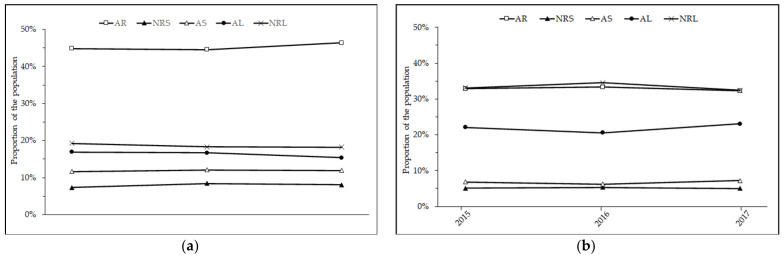
Trends in adherence to NSF sleep duration recommendations from 2015 to 2017: (**a**) on weekdays; (**b**) at weekends. AR—appropriate recommended duration; AS—appropriate but short; AL—appropriate but long; NRS—not recommended: too short; NRL—not recommended: too long.

Figure 2 shows the trends in sleep duration as they map onto the NSF recommendations on weekdays and at weekends from 2015 to 2017. There were no significant differences over the three-year period. In Table 6, we show the association between sociodemographic variables and sleep duration recommendations. All sociodemographic variables were significantly associated with sleep duration within the recommended guidelines (all *p*s < 0.001). Specifically, the likelihood of sleeping for an appropriate duration according to NSF recommendations increased with increasing educational attainment. This trend was also similar for increasing family income (Table 6). Sleep duration within the appropriate range was more likely for those with children than those without. Likewise, employed people and married people independently were the most likely to sleep for an appropriate duration.

**Figure 3:**
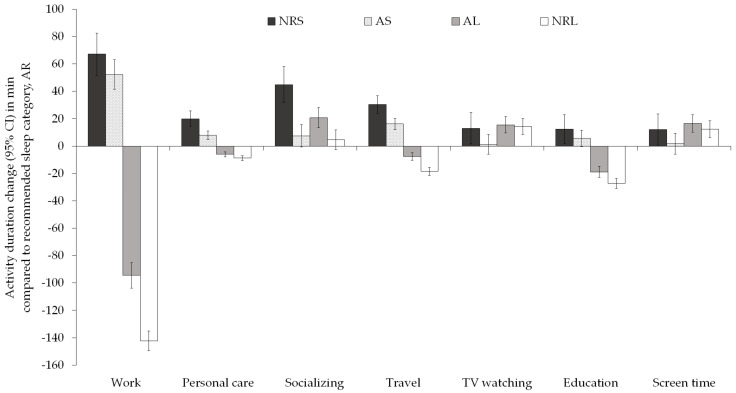
Adjusted deviation of duration of waking activity between nonadherence sleep duration categories (NRS, AS, AL, NRL) and the recommended sleep duration category (AR). AR—appropriate recommended duration; AS—appropriate but short; AL—appropriate but long; NRS—not recommended: too short; NRL—not recommended: too long.

We calculated the adjusted deviation in the duration of waking activities of respondents in each of the four nonaligned categories from the AR category in a weighted multiple linear regression. Regression models were adjusted for age, sex, ethnicity, educational attainment, children in household, marital status, family income, and employment status. Figure 3 shows seven waking activities where the adjusted deviation of duration of waking activity between the AR and at least one of the other sleep duration categories exceeded 15 min. Work was the waking activity with the largest adjusted change in duration between the sleep recommendation categories. Specifically, respondents whose sleep duration fell below the recommendations (NRS and AS), respectively, worked 67 min (95% CI: 52; 83 min) and 52 min (95% CI: 41; 63 min) longer than those who slept for the recommended sleep durations (*p*s < 0.001). Conversely, those with longer sleep duration, whether within the recommendations or not (AL or NRL), respectively worked 94 min (95% CI: -104; -85 min) and 142 min (95% CI: -150; -135 min) less than those in the AR category (*p*s < 0.001). A similar pattern of association was observed for travel time, personal care, and education—the population in the NRS and AS sleep duration categories spent more time on these activities, whereas those in the AL and NRL categories spent significantly less time compared to the AR category. Participants in the NRS category consistently exchanged sleep time for each of the seven waking activities presented in Figure 3 (all *p*s < 0.05).

When the multiple linear regression models were restricted to data collected on weekdays (Table 7), change in the duration of waking activities between at least one of the nonaligned categories compared to the AR category exceeding 15 min was recorded for the same seven activities (work and work-related activities; socialising, relaxing, and leisure; TV watching; screen time; travel; personal care; and education). Although the NRL group worked on average 124 min (95% CI: -135; -114 min) less than the AR category on weekdays, there was no significant difference in the adjusted time spent on socialising, relaxing, and leisure activities or watching TV in comparison to the AR category. Further, those in the NRL category spent significantly less time on travelling, personal care, and household activities (16 min, 9 min, and 10 min, respectively) compared to the AR category. By contrast, those in the NRS category spent significantly more time (27 min, 20 min, and 8 min) on travelling, personal care, and household activities on weekdays, respectively, compared to the AR category. Adjusted weekday screen time was 17 min (95% CI: 4; 29 min) higher for those in the NRS, and 12 min (95% CI: 3; 21 min) higher for those in the AL sleep duration categories compared to the reference sleep duration category.

Table 8 shows the results of weighted multiple linear regressions for the relationship between change in duration of each of 19 waking activities and sleep adherence categories at weekends. The waking activities which were mostly exchanged for sleep time at weekends were not dissimilar to those exchanged for sleep on weekdays. However, at weekends, time spent watching TV and screen devices in general did not significantly differ. Notably, the unadjusted time spent watching TV and using screens in general was approximately 50 min higher at weekends than on weekdays (Table S1).

There was a significant adjusted change in the duration of sports, exercise, and recreation between different sleep duration categories and the AR category. Specifically, respondents in the AS spent 10 min (95% CI: 2; 19 min) more on sports, exercise, and recreation than the AR group, whereas those in the AL and NRL categories spent significantly less time (-6 min (95% CI: -9; -3 min) and -7 min (95% CI: -11; -4 min), respectively) on sports, exercise, and recreation.

## 4. Discussion

Our findings show that less than half of the US population met the number of hours of sleep recommended in the NSF age-appropriate guidelines. Sleep duration among the US population mapped onto the NSF recommendations mostly on weekdays (among 45%) rather than at weekends (among 33%). Further, after controlling for sociodemographic variables, time spent on each of the following waking activities: work, personal care, socialising, relaxing, leisure, travel, TV watching, education, and screen time was inversely associated with sleeping the recommended number of hours, in general. These waking activities remained significantly associated with sleep duration when analysis was restricted to weekdays. At weekends, however, all but education remained significantly associated (by at least a 15 min difference) with sleep duration outside of the recommended number of hours. In addition, time spent on household activities at weekends was associated with sleep duration.

To our knowledge, this is the largest study using time-use survey data from a population representative sample in the US to describe reported sleep duration in relation to NSF age-specific recommendations among adults. Sleep duration in relation to recommendations in adult populations is rather limited as the NSF guidelines were published only five years ago. We show that about 60% of the US adult population’s sleep duration does not map onto the NSF recommendations on sleep duration when age-specific thresholds are applied. Sleeping duration outside of the recommendations was more common on weekdays than at weekends. Adding to the debate about global decline in sleep duration [23,24], this study reveals that the proportion of people sleeping longer than the recommended duration exceeds those sleeping less than the recommended duration even on weekdays. We show that the disparity between weekday and weekend sleep habits previously reported [25,26] is also evident at the population level. Longer sleep duration at weekends relative to weekdays may be explained by either later wake times to catch up on lost sleep during workdays [27] or a combination of later sleep time and later wake time at weekends. The former, i.e., attempted catch-up sleep at weekends, has been shown to be associated with an increased risk of poor health-related quality of life and anxiety/depression [28], while the latter, often referred to as social jetlag, has been linked with an increased risk of obesity, type 2 diabetes mellitus, and impaired metabolic control in noncommunicable diseases [29,30,31,32].

In the US population, time spent on waking activities—time spent on work, travel, personal care, socialising, relaxing, leisure, watching TV, and education—differed for those who slept the recommend number of hours and those who did not. Thus, the kinds of activities that are traded for sleep among the United States population have not changed over the past decade [17]. Using ATUS data from 2003 to 2005, Basner et al. [17] identified the same activities to be associated with sleep time (in hour intervals). However, in the present study, work and travel remained the only activities for which the linear gradient in its duration across the various sleep duration categories was significant both on weekdays and at weekends. We were unable to compare the observed consistent gradients with the findings of Basner et al. [17], who reported a similar declining gradient in the duration of waking activities, with increasing sleep time due to the use of different sleep duration categories and the absence of the level of significance for each individual sleep category in relation to the reference category. Similarly, a recent research study of daily time use among the UK adolescent population revealed that more time spent on personal care, travel, and education was associated with an increased risk of short sleep [21]. Nevertheless, the trends reported in the present study, including the lack of any visible gradient in TV watching across the various sleep categories, remain consistent with previous findings [17].

A strength of our study is that we directly mapped reported sleep duration to the NSF sleep recommendations using nationally representative data. The use of the age-specific sleep recommendation categories permitted the standardisation of sleep duration. In addition, we accounted for potential confounding variables in our regression analysis. The ATUS uses a probabilistic sampling technique in which every eligible individual has equal opportunity of being a participant, making the sample representative of the US teenage and adult population. Oversampling of minority ethnic groups and data collection during weekends were corrected with sampling weights provided by the ATUS. Further, the use of survey-specific statistical analysis permitted the estimation of population parameters rather than sampling statistics; thus, findings from this study can be extrapolated to the US population.

A limitation of this study is the cross-sectional nature of the data which does not allow for temporal and causal associations to be made between adherence to sleep recommendation and time spent on waking activities. The 24 h recall method used in collecting activity data for the ATUS does not take into account any prior activities (e.g., sleep deprivation on previous day) that could have influenced the time spent on activities during the interview period, thus opening the possibility of reverse causation. Although relatively low, recall bias remains an issue in the retrospective data collection format used in the ATUS. Further, the response rate of eligible participants in the ATUS was low for all three years, although this is not different from the ATUS response rate in earlier years. Investigations conducted on earlier cohorts revealed that nonrespondents were more likely to be weakly integrated in their communities [33]. A recent evaluation of the association between nonresponse propensity and the quality of the ATUS data revealed that ATUS nonrespondents had more missing data and rounded their responses in the parent survey, CPS, from which the ATUS sample is drawn [34]. Thus, data provided by ATUS respondents are more likely to provide an accurate picture even in the absence of data from the nonrespondents. In addition, secondary activities (i.e., activities occurring simultaneously with others) or activities of other household members were not recorded in the ATUS, so emphasis was only placed on each respondent’s choice of primary activity. In this article, sleep duration was computed from total sleep time during the day and as a result includes daytime napping. Whereas this may be a limitation, it permits the inclusion of shift workers whose nighttime sleep duration may not be reflective of their total sleep time. Additionally, the data are limited to a single 24 h recall; however, more precise measures such as polysomnography data are not feasible for such large population-based surveys. While the NSF guidelines provide a good indication of sleep duration recommendations, the data from which these recommendations are drawn may have often not included daytime naps for older adults, hence explaining the lower sleep duration expected for this group. In addition, results on screen use should be interpreted carefully since ATUS questionnaires do not accurately capture portable screen use. As a result, the measure of screen use in this paper is heavily indicative of television use rather than other commonly used screen devices, such as smartphones, and tablets which have been shown to be associated with poor sleep [35,36]. Further, other direct factors that may influence sleep times, such as exposure bright light, which have a direct influence on the physiological onset of sleep [27,37,38,39] and could mediate time spent on some waking activities, were also not considered in this study.

The present study has implications in promoting a balance between time spent on waking activities, particularly work-related activities, and time spent sleeping. While management of sleep and waking activities will vary greatly across professions and age groups, the ability to maintain healthy levels of sleep remains a problem in the current 24 h society [22,40,41].

## 5. Conclusions

This study shows that on a typical day, people whose sleep is very short and below the NSF recommendations spend more time on work, socialising, relaxing and leisure, personal care, and travel, whereas those whose sleep is above the NSF recommendations spend less time on all these activities both on weekdays and at weekends. This study shows that less than half of the US population adheres to the recommended sleep duration for their age. A significant portion of the population “catch up” on sleep deficits at weekends, warranting further study of the potential health implications of this behaviour.

## Figures and Tables

**Table 1:** The National Sleep Foundation’s age-specific guidelines for sleep duration in hours (h).

Age	Appropriate, Recommended Duration (AR)	Appropriate But Short (AS)	Appropriate But Long (AL)	Not Recommended: Too Short (NRS)	Not Recommended: Too Long (NRL)
New-borns					
0–3 month	14–17	11–<14	>17–19	<11	>19
Infants					
4–11 month	12–15	10–<12	>15–18	<10	>18
Toddlers					
1–2 years	11–14	9–<11	>14–16	<9	>16
Pre-schoolers					
3–5 years	10–13	8–<10	>13–14	<8	>14
School-aged children					
6–13 years	9–11	7–<9	>11–12	<7	>12
Teens					
14–17 years	8–10	7–<8	>10–11	<7	>11
Young adults					
18–25 years	7–9	6–<7	>9–11	<6	>11
Adults					
26–64 years	7–9	6–<7	>9–10	<6	>10
Older adults					
≥65 years	7–8	5–<7	>8–9	<5	>9

AR—appropriate recommended duration; AS—appropriate but short; AL—appropriate but long; NRS—not recommended: too short; NRL—not recommended: too long. Adapted with permission from Hirshkowitz, Whiton, Albert, et al. National Sleep Foundation’s sleep time duration recommendations: methodology and results summary. Sleep Health. 2015;1(1):40–43. doi:10.1016/j.sleh.2014.12.010an [15].

**Table 2:** Configuration of the American Time Use Survey (ATUS) database from 2015 to 2017.

Year	Designated Sample, *N*	ATUS Respondents *N* (%)	Men *N* (%)	Weekdays ^1^ *N*	Weekends *N*
2015	23,281	10,905 (46.8)	4778 (43.8)	5475	5430
2016	23,254	10,493 (45.5)	4670 (44.5)	5327	5166
2017	23,303	10,223 (43.9)	4642 (45.4)	5059	5144
Total	69,838	31,621 (45.3)	14,090 (44.6)	15,881	15,740

^1^ Includes holidays that fell on weekdays. Designated sample size was calculated from ATUS case file data and excluded noneligible participants. The ATUS response rate was calculated using the American Association for Public Opinion Research’s (AAPOR’s) formula as follows: Response rate = C/(C + R + NC + O + UE); where C = Completes (complete or sufficient partial interview), R = Refusals, NC = Noncontact (uncompleted callbacks; never contacted), O = Other (respondent absent, ill, or hospitalised; language barrier, etc.), and UE = Unknown eligibility (phone number incorrect for household, unconfirmed number, etc.).

**Table 3:** Sociodemographic characteristics of ATUS respondents 2015–2017.

Characteristics	Respondents, *N*	Weighted Percentage (95% CI)
Age (years)		
17–20	1515	8.1 (8.1; 8.1)
20–34	5889	25.2 (25.1; 25.3)
35–49	8368	23.6 (23.5; 23.6)
50–64	8106	24.4 (24.4; 24.5)
≥65	7287	18.7 (18.6; 18.7)
Sex, Male	13,893	48.3 (48.2; 48.4)
Race/ethnicity		
White	20,138	65.7 (65.3; 66.1)
Black	4471	11.9 (11.9; 12.0)
Hispanic	4757	16.1 (16.1; 16.2)
Asian	1246	4.4 (4.1; 4.8)
Other	553	1.9 (1.7; 2.0)
Highest level of education		
Less than high school	3966	15.6 (15.2; 16.0)
High school graduate	12,889	44.1 (43.5; 44.7)
College graduate	9892	28.6 (28.1; 29.2)
Masters or higher	4418	11.7 (11.3; 12.1)
Children in Household		
No child	18,407	61.9 (61.7; 62.1)
One or more children	12,758	38.1 (37.9; 38.3)
Marital status		
Married	15,197	52.2 (51.6; 52.7)
Divorced/Separated	5392	11.5 (11.1; 11.9)
Widowed	2886	5.5 (5.2; 5.7)
Never married	7690	30.9 (30.4; 31.4)
Family income		
<USD 50,000	14,626	42.9 (42.2; 43.6)
USD 50,000 to <100,000	9441	31.9 (31.2; 32.7)
≥USD 100,000	7098	25.2 (24.5; 25.8)
Employment		
Full-time student	1674	9.0 (8.5; 9.4)
Employed	17,978	57.3 (56.7; 58.0)
Unemployed	736	2.4 (2.2; 2.7)
Not in Labour Force/Retired	10,777	31.3 (30.8; 31.8)

ATUS—American Time Use Survey; CI—confidence interval.

**Table 4:** Weighted average sleep duration (standard error) on weekdays and at weekends by year and sex.

		Year of Survey			
		2015	2016	2017	Total
Weekday	Men	8.46 (0.06)	8.30 (0.06)	8.34 (0.06)	8.36 (0.03)
	Women	8.58 (0.05)	8.55 (0.05)	8.55 (0.05)	8.56 (0.03)
	All	8.52 (0.04)	8.43 (0.04)	8.45 (0.04)	8.46 (0.02)
Weekend	Men	9.27 (0.06)	9.39 (0.06)	9.26 (0.05)	9.31 (0.03)
	Women	9.36 (0.05)	9.39 (0.05)	9.38 (0.05)	9.38 (0.03)
	All	9.31 (0.04)	9.39 (0.04)	9.32 (0.03)	9.34 (0.02)

**Table 5:** Number (weighted percentage) of respondents adhering to NSF sleep age-specific recommendations.

		AR	AS	AL	NRS	NRL
Weekday	Men	3186 (45.8)	989 (13.2)	968 (15.0)	629 (9.0)	1181 (17.2)
	Women	3841 (44.8)	975 (10.7)	1460 (17.6)	605 (7.0)	1797 (19.9)
	All	7027 (45.3)	1964 (11.9)	2428 (16.3)	1234 (8.0)	2978 (18.6)
Weekend	Men	2363 (33.5)	514 (7.2)	1436 (21.4)	397 (5.7)	2230 (32.2)
	Women	2744 (32.2)	616 (6.4)	1902 (22.4)	392 (4.6)	2940 (34.4)
	All	5107 (32.8)	1130 (6.8)	3338 (21.9)	789 (5.2)	5170 (33.3)

NSF—United States National Sleep Foundation; AR—appropriate recommended duration; AS—appropriate but short; AL—appropriate but long; NRS—not recommended: too short; NRL—not recommended: too long.

**Table 6:** Sociodemographic factors associated with adherence of NSF sleep duration recommendation.

	Weighted Proportion, %					
Variables	AR	AS	AL	NRS	NRL	*p*
Age (years)						
17–20	39.0	10.0	17.2	7.1	26.7	<0.0001
20–34	42.2	8.1	21.1	7.3	21.3	
35–49	48.5	11.5	14.7	8.3	17.0	
50–64	49.7	11.9	13.2	9.0	16.3	
≥65	23.2	10.4	24.2	3.0	39.2	
Sex						
Male	42.2	11.5	16.8	8.0	21.5	<0.0001
Female	41.2	9.5	19.0	6.3	24.1	
Race/ethnicity						
White	43.8	10.1	18.5	6.7	20.8	<0.0001
Black	31.1	12.8	15.2	11.3	29.5	
Hispanic	41.0	9.8	17.1	6.2	26.0	
Asian	42.6	10.2	19.4	5.2	22.6	
Other	37.0	11.3	18.2	10.4	23.1	
Educational attainment						
Less than high school	35.7	9.6	16.9	6.9	30.9	<0.0001
High school graduate	38.8	10.0	19.2	7.2	24.7	
College graduate	45.8	11.0	17.1	7.5	18.6	
Masters or higher	50.5	11.8	16.3	6.2	15.2	
Children in Household						
No child	39.6	9.9	19.0	6.6	24.9	<0.0001
One or more children	45.0	11.2	16.2	8.1	19.5	
Marital status						
Married	44.7	11.0	17.4	6.9	20.1	<0.0001
Divorced/Separated	40.3	10.8	15.9	7.9	25.1	
Widowed	26.4	11.8	19.0	4.3	38.4	
Never married	39.9	9.1	19.4	7.7	23.9	
Family income						
<USD 50,000	35.6	9.5	18.7	7.0	29.1	<0.0001
USD 50,000–<100,000	42.7	11.0	18.7	7.0	20.6	
≥USD 100,000	50.7	11.3	15.5	7.5	15.0	
Employment						
Full-time student	38.7	7.9	22.8	7.4	23.2	<0.0001
Employed	48.0	12.0	15.2	8.0	16.8	
Unemployed	36.1	8.2	18.9	9.5	27.2	
Not in Labour Force/Retired	31.4	8.5	21.5	5.3	33.3	

NSF—United States National Sleep Foundation; AR—appropriate recommended duration; AS—appropriate but short; AL—appropriate but long; NRS—not recommended: too short; NRL—not recommended: too long.

**Table 7:** Relationship between daily time spent on each of the waking activities (in minutes) and adherence to NSF sleep duration recommendation on weekdays (*N* = 15,631).

	NSF Sleep Recommendation Categories				
	Duration (95% CI)	Change in Waking Activity Compared to Reference Category (AR) in min (95% CI)			
Waking Activity	AR	NRS	AS	AL	NRL
Work and work-related activities	450.6 (435.3; 465.8)	+53.7 (37.3; 69.7) ^#^	+41.3 (29.1; 53.5) ^#^	−73.0 (−84.6; −61.3) ^#^	−124.3 (−134.7; −113.9) ^#^
Socialising, relaxing, and leisure	218.9 (206.1; 231.6)	+47.7 (32.9; 62.6) ^#^	+7.9 (−1.5; 17.4)	+10.8 (0.9; 20.7) *	−8.4 (−18.7; 1.8)
TV watching	142.7 (131.6; 153.9)	+17.1 (4.2; 29.9) ^‡^	+4.2 (−4.1; 12.4)	+9.3 (1.2; 17.4) *	+4.4 (−4.4; 13.3)
Screen time	152.6 (141.2; 164.0)	+16.6 (4.0; 29.3) *	+4.4 (−4.4; 13.1)	+12.1 (3.1; 21.1) ^‡^	+2.3 (−7.1; 11.6)
Travel	73.9 (69.1; 78.7)	+26.8 (20.0; 33.5) ^#^	+16.2 (11.5; 20.8) ^#^	−7.8 (−11.5; −4.2) ^#^	−15.9 (−20.4; −11.5) ^#^
Personal care (excluding sleep)	33.6 (29.5; 37.7)	+19.5 (12.7; 26.4) ^#^	+7.8 (14.1; 11.5) ^#^	−7.3 (−9.7; −4.9) ^#^	−9.4 (−11.9; −6.9) ^#^
Household activities	71.2 (61.9; 80.5)	+ 8.2 (0.4; 16.1) *	−2.1 (−8.5; 4.4)	+10.0 (2.1; 17.8) *	−10.1 (−17.2; −2.9) ^‡^
Eating and drinking	60.7 (57.5; 63.9)	+1.5 (−2.8; 5.9)	−1.0 (−3.4; 1.5)	−0.8 (−3.6; 2.0)	−3.8 (−6.0; −1.6) ^‡^
Sport, exercise and recreation	15.7 (12.5; 18.9)	+2.8 (−1.2; 6.8)	+1.1 (−2.2; 4.4)	+1.1 (−1.9; 4.1)	−1.6 (−4.0; 0.8)
Consumer purchases	11.8 (9.0; 14.6)	+0.7 (−2.1; 3.5)	+0.6 (−1.6; 2.8)	+2.0 (−0.6; 4.6)	−1.0 (−3.6; 1.3)
Education	−3.9 (−9.9; 2.2)	+15.6 (3.2; 28.0) *	+5.9 (−0.6; 12.3)	−21.8 (−27.5; −16.0) ^#^	−31.6 (−37.4; −25.7) ^#^
Care for household members	−2.3 (−6.8; 2.1)	+2.6 (−1.8; 7.0)	+3.4 (−0.2; 6.9)	−1.7 (−5.3; 1.9)	−6.6 (−9.5; −3.8) ^#^
Care for nonhousehold members	8.7 (5.8; 11.5)	−0.2 (−2.8;2.4)	+1.5 (−1.1; 4.0)	−0.6 (−2.9; 1.7)	−1.8 (−4.2; 0.6)
Religious and spiritual activities	2.1 (−0.2; 4.4)	−0.1 (−2.2; 1.9)	+0.3 (−1.4; 2.0)	−0.9 (−2.2; 0.5)	−1.8 (−3.2; −0.5) ^‡^
Volunteer activities	4.4 (1.5; 7.3)	+3.5 (−0.3; 7.3)	+2.4 (−0.4; 5.1)	−0.5 (−3.2; 2.3)	−4.1 (−6.0; −2.1) ^#^
Telephone calls	0.5 (−1.0; 2.0)	+0.1 (−2.0; 2.3)	+1.0 (−0.6; 2.7)	−1.8 (−2.9; −0.7) ^‡^	−1.9 (−3.2; −0.7) ^‡^
Household services	1.4 (0.5; 2.3)	−0.2 (−0.9; 0.5)	−0.4 (−1.2; 0.5)	−0.2 (−0.8; 0.3)	−0.4 (−1.0; 0.2)
Professional/personal care services	2.2 (0.7; 3.7)	+2.1 (−0.4; 4.7)	+0.8 (−1.0; 2.5)	+0.3 (−1.1; 1.7)	+1.6 (0.1; 3.1) *
Government service and civic duties	0.1 (0.0; 0.2)	0.0 (−0.3; 0.3)	−0.1 (−0.2; 0.1)	0.0 (−0.2; 0.1)	0.1 (−0.2; 0.3)

* *p* < 0.05; ^‡^ *p* < 0.01; ^#^ *p* < 0.001; All models were adjusted for age, sex, ethnicity, educational attainment, children in household, marital status, family income, and employment status. The intercept of each adjusted linear regression model (shown in the AR category) indicates the average time spent on a waking activity at weekends for a white male respondent in the recommended sleep duration category, age 35–49 years, high school graduate, married, with no child, employed, and household income of USD 50,000–100,000.

**Table 8:** Associations between daily time spent on waking activities (in minutes) and sleeping duration according to NSF guidelines at weekends (*N* = 15,534).

	NSF Sleep Recommendation Categories				
	Duration (95% CI)	Change in waking activity compared to reference category (AR) in min (95% CI)			
Waking Activity	AR	NRS	AS	AL	NRL
Work and work-related activities	140.2 (126.6; 153.8)	+97.4 (72.2; 122.7) ^#^	+46.3 (28.7; 63.9) ^#^	−40.2 (−48.8; −31.5) ^#^	−60.5 (−67.1; −53.9) ^#^
Socialising, relaxing, and leisure	356.6 (341.7; 371.6)	+43.8 (15.1; 72.4) ^‡^	+29.7 (11.9; 47.5) ^‡^	−8.0 (−18.1; 2.0)	−35.3 (−45.2; −25.4) ^#^
TV watching	218.4 (203.9; 232.9)	+3.7 (−19.5; 27.0)	+1.9 (−15.3; 19.1)	+2.8 (−6.3; 11.9)	−0.5 (−8.9; 7.9)
Screen time	228.3 (213.9; 242.8)	+1.5 (−22.4; 25.4)	+4.5 (−13.7; 22.7)	+0.2 (−9.0; 9.4)	−2.7 (−11.2; 5.8)
Travel	79.1 (73.1; 85.1)	+44.3 (28,7; 60.0) ^#^	+17.1 (8.5; 25.7) ^#^	−8.9 (−13.0; −4.9) ^#^	−24.0 (−27.7; −20.2) ^#^
Personal care (excluding sleep)	30.2 (26.7; 33.6)	+21.5 (12.3; 30.7) ^#^	+8.0 (1.8; 14.2) *	−2.5 (−5.0; 0.0) ^#^	−6.6 (−8.9; −4.3) ^#^
Household activities	141.7 (130.8; 152.6)	−9.7 (−23.3; 3.9)	−9.6 (−21.1; 1.9)	−5.0 (−13.0; 3.0)	−26.9 (−33.1; −20.7) ^#^
Eating and drinking	71.7 (67.7; 75.7)	−0.4 (−6.8; 6.1)	−0.7 (−4.9; 3.4)	0.0 (−2.6; 2.6)	−4.5 (−6.9; 2.1) ^#^
Sport, exercise and recreation	32.3 (27.0; 37.5)	+7.2 (−2.6; 17.1)	+10.1 (1.5; 18.8) *	−5.9 (−9.2; −2.6) ^‡^	−7.4 (−10.9; −3.9) ^#^
Consumer purchases	30.7 (26.9; 34.6)	−3.8 (−9.0; 1.4)	−0.9 (−5.4; 3.6)	−1.4 (−4.4; 1.6)	−5.3 (−7.8; −2.8) ^#^
Education	4.3 (−0.5; 9.1)	−0.7 (−10.4; 0.1)	−1.3 (−7.0; 4.2)	−5.8 (−9.9; −1.7) ^‡^	−8.2 (−11.9;−4.4) ^#^
Care for household members	9.5 (5.8; 13.2)	+6.5 (−1.9; 14.9)	−2.4 (−6.8; 2.0)	−3.7 (−6.6; −0.7) *	−11.6 (−14.0; −9.2) ^#^
Care for nonhousehold members	12.1 (8.5; 15.7)	−1.8 (−6.0; 2.4)	−0.2 (−3.7; 3.2)	−1.9 (−4.4; 0.7)	−4.1 (−6.6; −1.6) ^‡^
Religious and spiritual activities	15.3 (11.1; 19.6)	−5.0 (−9.8; −0.2) *	−4.1 (−8.4; 0.1)	−0.7 (−3.5; 2.1)	−5.1 (−7.4; −2.8) ^#^
Volunteer activities	9.4 (6.2; 12.6)	5.0 (−1.5; 11.4)	2.3 (−1.6; 6.3)	−2.9 (−5.4; −0.3) *	−6.7 (−8.6; −4.9) ^#^
Telephone calls	0.7 (−0.8; 2.1)	+4.6 (−2.0; 11.1)	+1.6 (−0.5; 3.6)	−0.8 (−2.0; 0.4)	−2.0 (−3.1; −0.8) ^‡^
Household services	0.4 (0.0; 0.8)	0.0 (−0.7; 0.6)	−0.1 (−0.6; 0.4)	−0.3 (−0.6; 0.1)	−0.2 (−0.6; 0.1)
Professional/personal care services	1.9 (0.8; 3.0)	+2.1 (−0.2; 4.5)	−0.5 (−1.6; 0.7)	−0.5 (−1.5; 0.5)	−0.9 (−1.7; −0.1) *
Government service and civic duties	0.0 (−0.2; 0.2)	0.0 (−0.3; 0.3)	−0.1 (−0.2; 0.1)	0.0 (−0.2; 0.1)	0.1 (−0.2; 0.3)

* *p* < 0.05; ^‡^ *p* < 0.01; ^#^ *p* < 0.001; All models were adjusted for age, sex, ethnicity, educational attainment, children in household, marital status, family income, and employment status. The intercept of each adjusted linear regression model (shown in the AR category) indicates the average time spent on a waking activity at weekends for a white male respondent in the recommended sleep duration category, age 35–49 years, high school graduate, married, with no child, employed, and household income of USD 50,000–100,000.

## Data Availability

The American Time Use Survey dataset is publicly available on https://www.bls.gov/tus/#data (accessed on 22 October 2018) and contains no personally identifiable information.

## Supplementary Materials

The following are available online at https://www.mdpi.com/article/10.3390/ijerph18116154/s1, Table S1: Comparison of duration of waking activities between weekdays and weekends.
