# Supplementary material for: Sleep Duration and Waking Activities in Relation to the National Sleep Foundation’s Recommendations: An Analysis of US Population Sleep Patterns from 2015 to 2017
Source: Int J Environ Res Public Health. 2021 Jun 7;18(11):6154. doi: 10.3390/ijerph18116154 (PMC8201191; doi:10.3390/ijerph18116154)
Supplement: Supplementary file 1 [file ijerph-18-06154-s001.zip › ijerph-1190446-supplementary.pdf]

Table S1. Comparison of duration of wake activities between weekdays and weekends.

**Wake activity**	**Weighted average duration in minutes (standard error)**		***P***
	Weekdays	Weekends	
Work	251.6 (2.3)	72.2 (1.5)	<0.001
Socialising	251.4 (1.9)	341.4 (1.9)	<0.001
TV watching	150.9 (1.4)	199.4 (1.7)	<0.001
Screen time	162.7 (1.6)	210.6 (1.6)	<0.001
Travel	72.6 (0.7)	70.2 (0.8)	0.035
Personal care (excluding sleep)	47.6 (0.5)	44.0 (0.5)	<0.001
Household activities	101.8 (1.3)	128.5 (1.2)	<0.001
Eating and Drinking	61.4 (0.5)	70.0 (0.5)	<0.001
Sport, exercise and recreation	17.8 (0.5)	24.8 (0.7)	<0.001
Consumer purchases	18.3 (0.4)	29.3 (0.5)	<0.001
Education	33.2 (1.1)	12.2 (0.8)	<0.001
Care for household members	27.0 (0.5)	25.0 (0.5)	0.003
Care for non-household members	7.7 (0.3)	9.0 (0.4)	0.028
Religious and spiritual activities	4.8 (0.3)	18.8 (0.5)	<0.001
Volunteer activities	7.7 (0.4)	9.8 (0.5)	0.003
Telephone calls	6.2 (0.2)	6.0 (0.3)	0.440
Household services	1.2 (0.1)	0.6 (0.1)	<0.001
Professional and special care services	6.2 (0.3)	2.3 (0.2)	<0.001
Government services and civic responsibilities	0.6 (0.2)	0.1 (0.0)	0.005
